# Acute Hemichorea in a Young Patient Secondary to Ischemic Infarction in the Right Lentiform Nucleus: A Case Report

**DOI:** 10.7759/cureus.86969

**Published:** 2025-06-29

**Authors:** Yee Mon Thu, Ali Altahiry, Christos Nikola, Bader Mohamed

**Affiliations:** 1 Stroke and Neurology, The Royal London Hospital, Barts Health NHS Trust, London, GBR

**Keywords:** acute hemichorea, acute ischaemic stroke, movement disorders and tremors, patent foramen ovale (pfo), stroke in the young

## Abstract

Chorea is a hyperkinetic movement disorder characterised by brief, unpredictable, dance-like involuntary movements involving multiple body parts. Hemichorea is its unilateral variant, affecting one side of the body. Both can arise from diverse aetiologies, including genetic, vascular, metabolic, autoimmune, drug-induced, and infectious causes. We report the case of a 31-year-old male with no significant past medical history who developed sudden-onset, left-sided hemichorea and homonymous hemianopia. Brain MRI demonstrated an acute right lentiform infarct, predominantly involving the globus pallidus. The patient improved markedly within 24 hours following the initiation of antiplatelet therapy. Further investigations revealed a Stage 2A-shunt patent foramen ovale, deemed the likely aetiology. This case underscores the importance of recognising hemichorea as a rare but possible manifestation of acute ischaemic stroke and highlights the need for comprehensive diagnostic evaluation in young patients presenting with atypical stroke symptoms.

## Introduction

Hemichorea is a rare hyperkinetic movement disorder characterised by irregular, non-rhythmic, and involuntary movements confined to one side of the body. It is often associated with dysfunction in the basal ganglia, a group of subcortical nuclei involved in motor control. The underlying pathophysiology typically involves an imbalance in the basal ganglia-thalamocortical circuitry, particularly impaired GABAergic transmission leading to excessive thalamocortical excitability [1,2].

While hemichorea can arise from various causes such as genetic (e.g., Huntington’s disease), autoimmune, infectious, metabolic (e.g., non-ketotic hyperglycaemia), neoplastic, or iatrogenic and vascular insults such as ischaemic or haemorrhagic strokes are significant aetiologies, especially in elderly populations [3,4].

However, the occurrence of hemichorea in young adults due to acute ischaemic stroke is distinctly uncommon. Overall, post-stroke movement disorders occur in fewer than 4% of cases, and hemichorea-hemiballismus is reported in fewer than 1% [5,6]. Lesions most frequently involve the subthalamic nucleus or basal ganglia, though cortical strokes have also been implicated [7,8]. In younger patients, especially those without conventional vascular risk factors, paradoxical embolism via a patent foramen ovale (PFO) is a well-recognised yet often overlooked cause of cryptogenic stroke [9,10].

This case report presents a 31-year-old male who developed sudden-onset hemichorea and left homonymous hemianopia due to an acute infarction in the right lentiform nucleus. The patient was ultimately found to have a PFO, highlighting the importance of evaluating young patients with stroke-like symptoms for atypical causes and the potential for full neurological recovery with prompt antiplatelet therapy.

## Case presentation

A 31-year-old male presented to the Accident & Emergency Department with a five-hour history of dizziness, blurred vision, and involuntary, repetitive, dance-like movements affecting the left upper and lower limbs. He denied limb or facial weakness, sensory disturbances, dysarthria, facial asymmetry, or loss of consciousness. There was no history of recent fever, joint pain, swelling, or cutaneous rash. His past medical history was unremarkable, with no prior use of medications, recreational drugs, or alcohol. There was no family history of stroke, movement disorders, or thrombophilia.

On neurological examination, he demonstrated a complete left homonymous hemianopia, with a National Institutes of Health Stroke Scale score of 2. Involuntary, irregular, non-rhythmic, choreiform movements were observed in the left upper and lower limbs. No clinical signs of deep vein thrombosis were noted on peripheral examination.

Laboratory investigations, including full blood count, platelet function tests, coagulation tests, routine biochemistry tests, thyroid function tests, vitamin B12, HbA1c and erythrocyte sedimentation rate, were largely unremarkable, aside from elevated lipid levels including total cholesterol and triglycerides (Table 1).

**Table 1:** Laboratory investigations showing all routine laboratory investigations are unremarkable apart from high serum total cholesterol and triglyceride.

General haematology	Patient’s value	Normal reference range
Haemoglobin	14.4g/dL	13.0–17.0 g/dL
White cell count	6.1 x 10^9/L	4–10 x 10^9^/L
Platelets	236 x 10^9/L	150–410 x 10^9^/L
Haematocrit	0.42	0.4–0.5
Red blood count	5.31 x 10^12/L	4.5–5.5 x 10^12^/L
Mean cell volume	83.8 fL	83–101 fL
Mean cell haemoglobin concentration	328 g/L	315–345 g/L
Neutrophil count	3.0 x 10^9/L	2–7 x 10^9^/L
Lymphocyte	2.6 x 10^9/L	1–3 x 10^9^/L
Monocyte	0.4 x 10^9/L	0.2–1 x 10^9^/L
Eosinophil	0.1 x 10^9/L	0–0.5 x 10^9^/L
Basophil	0.0 x 10^9/L	0–0.1 x 10^9^/L
Nucleated red blood cell count	0.0 x 10^9/L	0–0.2 x 10^9^/L
Immature granulocytes	0.0 x 10^9/L	0–0.3 x 10^9^/L
Erythrocyte sedimentation rate	5 mm/hour	1–10 mm/hour
Coagulation		
Prothrombin time	10.5 seconds	8.8–11.7 seconds
Prothrombin time/International normalized ratio	1.0	0.9–1.1
Activated partial thromboplastin time	26 seconds	21–29 seconds
Activated partial thromboplastin time ratio	1.0	0.9–1.1
Haematinics		
Ferritin serum	78 µg/L	30–400 µg/L
Vitamin B12 serum	316 ng/L	197–1,000 ng/L
Folate serum	4.3 µg/L	3.9–9.999 µg/L
General biochemistry		
Sodium serum	137 mmol/L	133–146 mmol/L
Potassium serum	4.1 mmol/L	3.5–5.3 mmol/L
Urea	4.6 mmol/L	2.5–7.8 mmol/L
Creatinine	100 µmol/L	59–104 mmol/L
Estimated glomerular filtration rate	(c) 76 mL/minute	
Total bilirubin serum	4 µmol/L	0–21 µmol/L
Alanine aminotransferase serum	20 U/L	0–41 U/L
Alkaline phosphatase serum	77 U/L	30–130 U/L
Total protein serum	72 g/L	60–80 g/L
Albumin serum	47 g/L	35–50 g/L
Calcium	2.37 mmol/L	
Adjusted calcium	2.29 mmol/L	2.2–2.6 mmol/L
Inorganic phosphate serum	1.01 mmol/L	0.8–1.5 mmol/L
Cholesterol serum	6.5 mmol/L (high)	0–5 mmol/L
Low-density lipoprotein cholesterol serum	1.6 mmol/L	0–2.6 mmol/L
High-density lipoprotein cholesterol serum	0.9 mmol/L	0.9–1.5 mmol/L
Triglycerides serum	4.61 mmol/L (high)	0–1.7 mmol/L
Cholesterol: high-density lipoprotein ratio serum	7.2	0–5
C-reactive protein serum	<1 mg/L	0–5 mg/L
Haemoglobin A1C	40 mmol/mol	20–41 mmol/mol
Endocrinology		
Free T4 serum	16.4 pmol/L	10.5–24.5 pmol/L
Thyroid-stimulating hormone serum	2.10 mU/L	0.27–4.2 mU/L
25-OH vitamin D serum	45 nmol/L	>50 nmol/L; sufficient for most people

Infection screen, including HIV, hepatitis B, hepatitis C serology, syphilis, and respiratory viral swabs, were negative (Table 2).

**Table 2:** Routine infection screen result.

Viral serology	Result
Hepatitis surface antigen qualitative (HBsAg)	Antigen not detected
Hepatitis core antibody qualitative (Anti-HBc)	Antibody not detected
HCV IgG qualitative	Antibody not detected
HIV 1 and 2 antibody qualitative	Antibody not detected
Syphilis enzyme immunoassay IgG/M	Antibody not detected
Coronavirus (2019-nCoV)	SARS-CoV-2 RNA not detected
RSV RNA qualitative	RNA not detected
Influenza A RNA qualitative	RNA not detected
Influenza B RNA qualitative	RNA not detected

**Figure 1:**
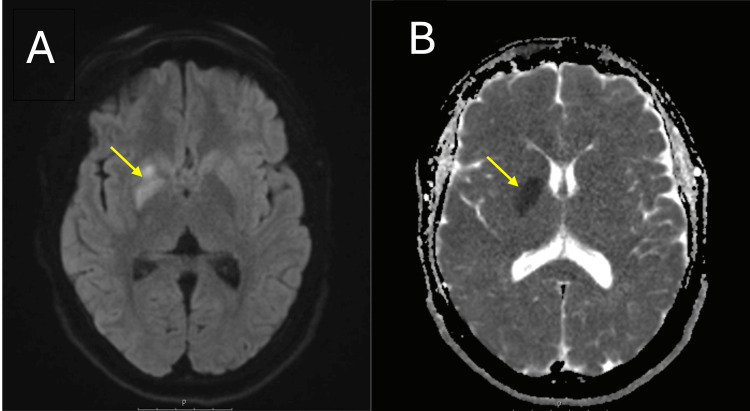
MRI of the head. (A) Diffusion-weighted imaging demonstrating restricted diffusion in the right lentiform nucleus, predominantly involving the globus pallidus, consistent with acute infarction. (B) Apparent diffusion coefficient map showing corresponding hypointensity, confirming true restricted diffusion.

ECG showed sinus rhythm, and transthoracic echocardiography was normal without evidence of cardiac thrombus. Initial CT of the head and CT angiogram of the aorta and intracranial vessels were normal. The patient was not eligible for thrombolysis due to being outside the therapeutic window and was not considered for mechanical thrombectomy as there was no evidence of a large intracranial artery occlusion. An MRI of the brain performed on the following day demonstrated an acute right lentiform infarct, predominantly involving the globus pallidus (Figure 1).

The patient was given a 300 mg loading dose of aspirin followed by dual antiplatelet therapy (aspirin 75 mg and clopidogrel 75 mg daily) for 21 days, followed by single antiplatelet therapy with long-term clopidogrel 75 mg. Atorvastatin 80 mg daily was initiated. The patient experienced complete symptom resolution within 48 hours and was discharged with outpatient follow-up.

Holter monitoring performed three weeks post-discharge revealed no arrhythmia. However, a bubble contrast echocardiography identified a Stage 2A shunt PFO, with a Risk of Paradoxical Embolism (RoPE) score of 8, suggesting a high likelihood of stroke attributable to PFO. The case was subsequently reviewed by the multidisciplinary stroke team to evaluate the potential need for PFO closure.

## Discussion

Hemichorea is an uncommon hyperkinetic movement disorder, typically presenting with irregular, involuntary, non-rhythmic movements predominantly affecting the distal extremities. It is most frequently associated with lesions in the basal ganglia, particularly the lentiform nucleus, subthalamic nucleus, and, less commonly, cortical regions [1,4,8]. The pathophysiology involves basal ganglia dysfunction, with impaired GABA transmission reducing thalamic inhibition and resulting in excessive thalamocortical excitation [4,5].

The differential diagnosis of hemichorea is broad and includes genetic conditions such as Huntington’s disease, autoimmune disorders (e.g., systemic lupus erythematosus, autoimmune encephalitis), neurodegenerative syndromes, infectious causes (e.g., Sydenham’s chorea, viral encephalitis), metabolic derangements (e.g., non-ketotic hyperglycaemia, hyponatraemia, hypocalcaemia), systemic disorders (e.g., renal or hepatic failure), endocrinopathies (e.g., thyroid dysfunction), nutritional deficiencies (e.g., thiamine, niacin), drug-induced etiologies (e.g., neuroleptics, dopamine antagonists), and exposure to toxins (e.g., heavy metals) [1,6]. In our case, the sudden onset of unilateral involuntary movements in a young adult, in the absence of systemic or metabolic abnormalities, raised suspicion for a vascular aetiology. MRI findings of an acute infarct in the right lentiform nucleus, particularly the globus pallidus, confirmed stroke-induced hemichorea. While stroke remains the most common vascular cause of hemichorea, other differentials such as metabolic abnormalities (e.g., non-ketotic hyperglycemia), autoimmune choreas, drug-induced dyskinesias, and infectious etiologies were considered and excluded in our workup. Additionally, neoplastic and paraneoplastic causes were not suggested by the clinical course or imaging findings. Nonetheless, we acknowledge a degree of diagnostic uncertainty, especially given the atypical presentation and the reversible nature of the symptoms. Recognition of this rare stroke presentation is vital, especially in younger patients presenting with isolated movement disorders. Prompt initiation of antiplatelet therapy was associated with rapid clinical improvement, emphasising the importance of early recognition and intervention. This case highlights the diagnostic superiority of MRI in detecting subcortical infarcts, particularly within the basal ganglia, which may not be visualised on initial CT scans. The sensitivity of diffusion-weighted imaging (DWI) in identifying acute ischaemic lesions underscores the necessity of MRI in patients presenting with isolated movement disorders when CT findings are inconclusive.

**Figure 2:**
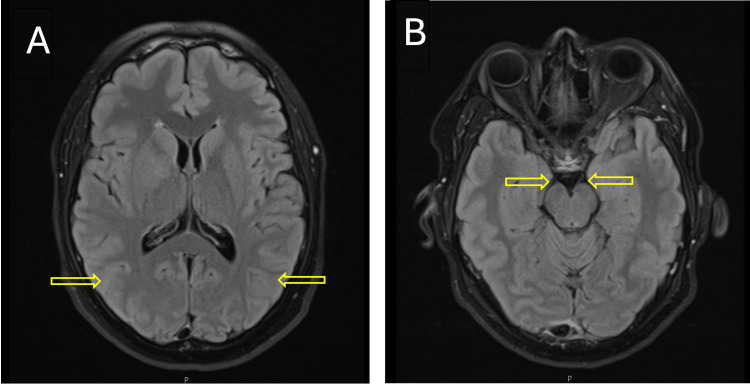
MRI of the head axial T2-weighted dark fluid MRI sequence. Axial T2-weighted dark fluid MRI sequence demonstrating no signal abnormalities in the occipital cortex or optic tracts. (A) No signal abnormalities in the occipital cortex bilaterally. (B) No signal abnormalities in the optic tracts bilaterally.

The globus pallidus receives its blood supply from penetrating branches of the anterior choroidal artery (a branch of the internal carotid artery) and the lateral lenticulostriate arteries from the middle cerebral artery. These small vessels are particularly susceptible to embolic occlusion, especially in cardioembolic strokes. In our case, the infarct’s location plausibly explains the patient’s hemichorea through disruption of basal ganglia-thalamocortical motor circuits. The concurrent left homonymous hemianopia may be attributed to involvement of adjacent structures such as the optic radiations or the posterior limb of the internal capsule. Importantly, axial T2-weighted dark fluid MRI sequence demonstrated no signal abnormalities in the occipital cortex or optic tracts (Figure 2). The absence of cortical or pre-geniculate involvement supports the hypothesis that the patient’s homonymous hemianopia may be secondary to subcortical involvement, likely affecting the optic radiations adjacent to the lentiform nucleus.

Additionally, this infarct pattern showed restricted diffusion on DWI with corresponding hypointensity on apparent diffusion coefficient, consistent with true cytotoxic oedema rather than DWI shine-through (Figure 1). However, the patient experienced complete symptom resolution within 48 hours, which raises the hypothesis of penumbral salvage, whereby timely or spontaneous reperfusion rescues functionally impaired but structurally viable tissue in the ischemic penumbra.

Recent literature highlights the broad spectrum of cerebral infarct locations that can result in hemichorea. While basal ganglia lesions remain the classical cause, cortical regions including the frontal, temporal, parietal, and insular lobes have also been implicated [2].

Additionally, a case series demonstrated hemichorea following temporal cortical infarctions [2], while another reported a case linked to isolated frontal cortical stroke [8]. These findings expand the neuroanatomical landscape of post-stroke movement disorders and suggest that lesions outside the basal ganglia may disrupt interconnected motor networks. While non-contrast CT remains a common first-line imaging modality for stroke, it may fail to detect small or deep infarcts in subcortical structures such as the basal ganglia. Given that these regions are frequently involved in hemichorea, this underscores the superior sensitivity of MRI in evaluating atypical stroke presentations and movement disorders. Our case reinforces the more classic association between basal ganglia infarction, specifically the globus pallidus and hemichorea, serving as a counterpoint to recent reports of cortical causes. This contrast underscores the diagnostic complexity and variability of post-stroke hyperkinetic presentations. These findings collectively highlight the need for clinicians to maintain diagnostic vigilance when confronted with acute unilateral hyperkinetic movements. Neuroimaging should extend beyond basal ganglia evaluation to include cortical and subcortical areas when the clinical picture is atypical.

Furthermore, in young adults without conventional vascular risk factors, the aetiology of stroke is often cryptogenic. In such cases, a comprehensive evaluation for cardioembolic sources is essential. One important consideration is the presence of a PFO, which has been recognised as a potential cause of paradoxical embolism in stroke patients [9,10]. In this case, bubble contrast echocardiography identified a Stage 2A shunt PFO. The patient’s RoPE score was 8, indicating a high probability that the PFO was causally related to the stroke. The RoPE score, which ranges from 0 to 10, incorporates factors such as patient age, infarct location, and the absence of traditional vascular risk factors to estimate both the likelihood of a pathogenic PFO and the potential risk of stroke recurrence.

We recognise that PFO-related strokes are typically embolic and often originate from peripheral venous sources. In this case, deep vein thrombosis (DVT) screening with lower limb Doppler ultrasonography was not performed, which limits our ability to confirm the embolic source. Additionally, thrombophilia screening (including Factor V Leiden, prothrombin gene mutation, protein C/S, and antithrombin III levels) was not completed during the acute phase but remains an important consideration in evaluating stroke in the young. These omissions reflect real-world limitations but also underscore the need for standardised evaluation protocols in cryptogenic stroke. Guidelines suggest that a minimum etiologic workup in young stroke patients should include brain and vascular imaging such as MRI, MR angiogram, CT, CT angiogram, cardiac evaluation with echocardiography (transthoracic echocardiogram ± bubble echocardiogram), continuous rhythm monitoring (e.g., Holter), screening for thrombophilia, autoimmune, metabolic, and infectious panels when indicated.

The incidence of hemichorea following stroke is rare. Hemichorea-hemiballismus occurs in fewer than 1% of all stroke patients, while overall movement disorders develop in approximately 1-4% of cases [5-7]. A large cohort study analysing over 5,000 patients found an incidence of post-stroke hemichorea of only 0.54% [4]. These figures underscore the clinical rarity of this presentation and support the importance of reporting such cases to improve recognition and understanding among clinicians.

Ultimately, this case highlights the importance of maintaining a high index of suspicion for stroke in patients with acute unilateral hyperkinetic movements. Neuroimaging should not be limited to the basal ganglia but should encompass cortical and subcortical structures. In younger patients, etiological evaluation should include a thorough search for potential sources of paradoxical embolism, particularly in the presence of a PFO. This should encompass cardiac investigations as well as venous studies, such as Doppler ultrasonography of the lower limbs to assess for DVT, as well as thrombophilia screening as a possible embolic source. Timely diagnosis and tailored management can lead to favourable outcomes even in these atypical stroke presentations.

## Conclusions

Hemichorea is an uncommon but clinically significant manifestation of acute ischaemic stroke, most often associated with lesions in the basal ganglia. While it is typically observed in older adults, this case illustrates that it can also occur in younger patients, sometimes in the absence of classic vascular risk factors. In such atypical presentations, comprehensive diagnostic evaluation, including neuroimaging and cardiovascular workup, may uncover underlying causes such as paradoxical embolism through a PFO. This case raises the hypothesis that stroke mechanisms in younger individuals may involve uncommon aetiologies and presentations, warranting further clinical attention and investigation. Recognising unilateral hyperkinetic movements as a potential, though rare, cerebrovascular symptom may contribute to timely diagnosis and appropriate secondary prevention. This case reinforces the importance of maintaining clinical vigilance, especially in atypical presentations, and highlights the role of multidisciplinary collaboration in managing stroke in young adults.
